# Multimorbidity Among Patients Receiving Home Care in Japan: Prevalence, Levels, and Association With Emergency Home Visits

**DOI:** 10.1002/jgf2.70170

**Published:** 2026-08-25

**Authors:** Gen Nakayama, Shoichi Masumoto, Yu Sun, Mitsuaki Ishii, Sayaka Nin, Takashi Inaba, Natsuki Kajikawa, Shuhei Hamada, Noriyuki Kimura, Jun Hamano

**Affiliations:** ^1^ Department of Primary Care and Medical Education, Institute of Medicine University of Tsukuba Tsukuba Japan; ^2^ Center for Supercentenarian Medical Research Keio University School of Medicine Tokyo Japan; ^3^ Kizuna Home Care Clinic Tokyo Japan; ^4^ Department of Family Medicine, General Practice and Community Health, Institute of Medicine University of Tsukuba Tsukuba Japan; ^5^ Department of General Medicine Tsukuba Central Hospital Ushiku Japan; ^6^ Department of Pharmacotherapy, Faculty of Pharmaceutical Sciences Hokkaido University of Science Sapporo Japan; ^7^ Department of Internal Medicine Kamisu Saiseikai Hospital Kamisu Japan; ^8^ Department of Palliative and Supportive Care, Institute of Medicine University of Tsukuba Tsukuba Japan

**Keywords:** home care services, homebound persons, house calls, multimorbidity, multiple chronic conditions, primary health care

## Abstract

**Background:**

Despite its clinical importance in primary care, the prevalence and levels of multimorbidity among patients receiving home‐based medical care remain poorly characterized, and its potential impact on emergency home visits is unclear. This study described the prevalence and levels of multimorbidity among patients receiving physician‐led home care in Japan and examined the association between multimorbidity level and emergency home visits.

**Methods:**

A multicenter cross‐sectional study was conducted in 2024 using physician‐completed questionnaires derived from medical records. Multimorbidity was defined as the presence of two or more chronic conditions. Based on prior literature, multimorbidity level was categorized as low (2–3 conditions), medium (4–5 conditions), or high (≥ 6 conditions). The primary outcome was the occurrence of an emergency home visit within the preceding month.

**Results:**

Among 506 included patients, 94.7% met criteria for multimorbidity, and 13.2% required an emergency home visit. In multivariable generalized linear mixed‐effects models with a logit link, higher multimorbidity levels were independently associated with increased odds of emergency home visits, with adjusted odds ratios of 2.16 (95% CI, 1.09–4.31) for the medium‐level group and 2.72 (95% CI, 1.19–6.19) for the high‐level group compared with the low‐level group.

**Conclusions:**

Multimorbidity was highly prevalent among Japanese patients receiving home care, exceeding prior domestic estimates while aligning with international reports. Higher levels of multimorbidity were associated with an increased likelihood of emergency home visits, suggesting that assessment of multimorbidity level may, if confirmed in future studies, inform risk stratification and policy planning for home‐based care services.

**Trial Registration:**

UMIN000056282

## 1 Background

Addressing complex health problems such as multimorbidity is a key feature of expert generalist practice [1]. Multimorbidity is commonly defined as the coexistence of two or more chronic conditions within an individual [2]. Patients living with multimorbidity experience numerous adverse outcomes, including increased mortality risk [3, 4, 5], reduced health‐related quality of life [6, 7], and functional disability [7, 8]. In response to these challenges, care for individuals with multimorbidity should emphasize person‐centered approaches that prioritize patient and caregiver values, minimize treatment burden, and ensure alignment of care with individual goals [9]. Among patients with multimorbidity, those who are housebound represent a particularly complex subgroup with substantial care needs [10]. Home‐based medical care has therefore been recognized as an effective strategy for addressing the clinical and functional complexity associated with multimorbidity [11, 12].

Despite its clinical importance, the prevalence of multimorbidity among patients receiving home care has not been fully characterized. A study conducted in Ontario, Canada, reported a prevalence of 92.1% [13], while a similar prevalence of 91.6% was observed in Berlin, Germany [14]. In contrast, a previous Japanese study of patients receiving home visits from general practitioners reported a substantially lower prevalence of 53.7% [15]. The limited number of investigations focused on home care populations, together with the considerable variability in reported prevalence, underscores the need for further research to clarify the burden of multimorbidity in this setting.

Multimorbidity is strongly associated with increased healthcare utilization, including prolonged hospital stays and higher healthcare expenditures [16]. Beyond its presence alone, greater multimorbidity burden is linked to substantially increased service use; for example, U.S. Medicare data indicate that individuals with high‐level multimorbidity experience approximately sevenfold more emergency department visits than those with low‐level multimorbidity [17]. Although no universal definition of multimorbidity level exists, prior studies have commonly operationalized higher levels using simple counts of chronic conditions, with thresholds of five or six or more conditions indicating high burden [13, 17, 18]. In home care populations, elevated multimorbidity levels have also been associated with more frequent emergency department visits among patients with dementia [19]. However, the relationship between multimorbidity level and emergency home visits, a core component of home‐based medical care, has not been previously examined.

Japan represents the most rapidly aging society globally and has implemented policies promoting physician‐led home care as part of a broader transition from hospital‐centered to community‐based healthcare delivery. Within this system, emergency home visits for acute illness or exacerbation of chronic disease are considered an essential function of home care services [20]. Continuous 24‐h on‐call availability further enhances access for patients and family caregivers in home‐based primary care [21]. Nevertheless, emergency home visits may impose substantial workload and resource burdens on physicians, highlighting the importance of identifying factors associated with their occurrence. Previous studies have linked emergency home visits to cancer status and specific medical procedures [22, 23, 24], yet the contribution of multimorbidity—particularly its level—remains insufficiently understood.

Accordingly, the objectives of the present study were twofold: first, to describe the prevalence and levels of multimorbidity among patients receiving physician‐led home care in Japan; and second, to examine the association between multimorbidity level and emergency home visits. Clarifying these relationships may support clinicians and policymakers in optimizing home care system design according to patients' clinical needs and risk profiles, and may also provide insights relevant to other countries confronting the healthcare challenges of population aging.

## 2 Methods

### 2.1 Study Design and Setting

A multicenter cross‐sectional study was conducted in 2024 among patients receiving physician‐led home visits. In Japan, home medical care is delivered through multidisciplinary collaboration involving physicians, as well as visiting nurses, rehabilitation therapists, and pharmacists. Physicians provide clinical assessment, ongoing medical management, and prescription of treatments during home visits for individuals who experience difficulty accessing facility‐based care.

Data collection was performed using a questionnaire survey administered across seven medical facilities providing physician home‐visit services in Tokyo and Ibaraki Prefecture. Five participating sites were community‐based clinics, and two were small‐ to medium‐sized local hospitals. The participating physicians were primary care physicians practicing in medical facilities associated with our university that offer family medicine training programs. One clinic was located in Tokyo, while six facilities were located in Ibaraki Prefecture. Tokyo represents a highly urbanized region with a dense concentration of healthcare resources, whereas Ibaraki Prefecture encompasses suburban and rural areas with comparatively limited medical infrastructure. Inclusion of facilities from both geographic contexts enabled capture of variation in healthcare delivery environments.

### 2.2 Participants and Data Collection

Eligible participants were patients who received physician home visits from the seven participating facilities between February and March 2024. Inclusion criteria comprised age ≥ 18 years and residence in a private home rather than a residential care facility. Exclusion criteria included initiation of home visits after February 2024 and the presence of unstable physical symptoms or a limited prognosis. The latter criterion was applied because this work was part of a larger project that collected data from both physicians and patients, and participation could place a burden on patients in unstable condition [25].

In the present study, all study variables were derived from physician‐completed questionnaires based on medical record review. Informed consent was obtained through distribution of written explanatory materials to patients and, when a family caregiver was present, to that caregiver as well; this allowed a caregiver to provide consent on behalf of patients with cognitive impairment or similar difficulty understanding the materials. When direct consent could not be obtained through either route, an opt‐out procedure was implemented. Use of medical record data was approved by the institutional review board of the affiliated university.

### 2.3 Measures

#### 2.3.1 Assessment of Multimorbidity

Multimorbidity was defined as the coexistence of two or more chronic conditions and was assessed using a structured two‐step procedure. First, attending physicians reported each patient's primary diagnosis—the condition prompting initiation of home care—using free‐text entry. Second, physicians identified coexisting chronic conditions from a predefined list of 18 categories. These 18 categories were developed with reference to the multimorbidity classification used in a previous study by Masumoto et al. [26] The same categories, in turn, were adapted from classifications used in previous nationwide studies in Japan [27, 28]. This approach was chosen to align with prior Japanese multimorbidity research where feasible. These categories included hypertension; diabetes; hyperlipidemia; cerebrovascular disease; cardiac disease (e.g., ischemic heart disease, atrial fibrillation); chronic respiratory disease (e.g., asthma, chronic obstructive pulmonary disease); digestive disease (e.g., gastroesophageal reflux disease, liver cirrhosis); kidney disease (e.g., chronic kidney disease); urologic disease (e.g., prostatic hypertrophy, neurogenic bladder); arthritis or rheumatism (e.g., osteoarthritis, rheumatoid arthritis); musculoskeletal conditions (e.g., disuse syndrome, lumbar spinal canal stenosis); fracture‐related conditions (e.g., femoral neck fracture); osteoporosis; dementia; neurological disease (e.g., Parkinson disease, epilepsy); mental disorders (e.g., depression, anxiety); endocrine disorders (e.g., thyroid disease); and malignancy. Disuse syndrome—commonly used in Japan to describe chronic functional decline resulting from prolonged inactivity—was categorized as a musculoskeletal condition because of its clinical relevance in home care populations.

Free‐text primary diagnoses were mapped to one of the 18 condition categories by a researcher with clinical expertise in geriatric medicine and primary care, with ambiguous classifications reviewed by a second researcher. Diagnoses that could not be appropriately categorized, such as sarcoidosis, were categorized as “other” and excluded from the chronic condition count, consistent with standard multimorbidity research approaches that rely on predefined condition lists. The exact category descriptions used in the questionnaire, including all illustrative examples listed for each of the 18 categories, and their correspondence to rubrics of the International Classification of Primary Care, second edition (ICPC‐2), are provided in Table S1.

#### 2.3.2 Independent and Outcome Variables

The primary independent variable was the level of multimorbidity, operationalized as the total number of chronic conditions. Consistent with prior literature [18], the level of multimorbidity was categorized into three groups: low (2–3 chronic conditions), medium (4–5 chronic conditions), and high (≥ 6 chronic conditions); patients with a single chronic condition were excluded from the analyses involving multimorbidity level as the independent variable.

The primary outcome was the occurrence of an emergency (unscheduled) home visit. In the Japanese home care system, scheduled home visits (regular home visits provided on a planned basis) are distinguished from emergency home visits, which are unscheduled visits made at the request of patients or their caregivers. Such visits are made for a range of reasons, with fever and death certification reported as common indications [15, 24]. Using a structured questionnaire, attending physicians reported whether the patient had required any emergency home visits during the preceding month, with responses recorded dichotomously as yes or no. The reasons for these visits and their clinical urgency were not assessed in the present study.

#### 2.3.3 Covariates

Based on previous studies [22, 23, 24], we selected the following variables as covariates: age, sex, presence of a primary diagnosis of malignant disease, use of home oxygen therapy or ventilatory support, presence of peripheral or central venous catheters, and presence of an indwelling urinary catheter or cystostomy. Although earlier research has demonstrated an association between long‐term care need level and emergency home visits [22, 24], this variable was excluded from the primary analytical model because it was considered a consequence of multimorbidity [29]. Similarly, use of home‐visit nursing services, which has also been associated with emergency home visits [23], was not incorporated into the primary model because it is largely determined by long‐term care need level [30].

Both variables were subsequently evaluated in sensitivity analyses to assess the robustness of the primary findings. Within Japan's long‐term care insurance framework, care‐need certification is categorized as “support need” (two levels) or “care need” (five levels). For analytical purposes, care‐need status was grouped into three categories: lower (support need levels 1–2 or not certified), middle (care need levels 1–3), and higher (care need levels 4–5). “Not certified” denotes individuals who had not applied for or had not received certification under the national long‐term care insurance system.

### 2.4 Statistical Analysis

Descriptive statistics were calculated to summarize patient characteristics, the number of chronic conditions, and the distribution of multimorbidity levels. Associations between multimorbidity level and emergency home visits were evaluated using generalized linear mixed‐effects models with a logit link, incorporating facility as a random intercept to account for clustering. Both unadjusted and adjusted models were estimated. The primary adjusted model included age, sex, presence of a primary diagnosis of malignant disease, use of home oxygen therapy or ventilatory support, presence of peripheral or central venous catheters, and presence of an indwelling urinary catheter or cystostomy, with all covariates treated as categorical variables. Missing data were handled using multiple imputation with 20 imputations based on fully conditional specification. Missing values in the outcome variable were imputed together with covariates; details and justification are provided in the Supporting Information. The intraclass correlation coefficient (ICC) was calculated from the facility‐level random intercept variance using the latent variable method and averaged across the 20 imputed datasets. All statistical analyses were performed using R version 4.5.2 (R Foundation for Statistical Computing, Vienna, Austria), specifically employing the *lme4* and *mice* packages.

### 2.5 Sensitivity Analyses

We conducted several sensitivity analyses to assess the robustness of the findings. First, we additionally adjusted for long‐term care need level and use of home‐visit nursing services to evaluate the stability of observed associations when including these potential mediators. Second, we performed a sensitivity analysis excluding disuse syndrome from the chronic condition count, recalculating the number of chronic conditions and the distribution of multimorbidity levels. Third, we modeled chronic condition count as a continuous variable using restricted cubic splines to examine the shape of the dose–response relationship without relying on the categorical cutoffs used in the primary analysis. Fourth, given the relatively low number of events per parameter in the primary model, we repeated the analysis using Firth's penalized likelihood method. Details of these analyses are provided in the Supporting Information.

## 3 Results

### 3.1 Participant Characteristics and Prevalence of Emergency Home Visits

Of the patients identified across the seven participating facilities, data were obtained for 506 (participant flow is shown in Figure S1). Participant characteristics and the prevalence of emergency home visits are summarized in Table 1. The study population consisted predominantly of older adults, with 68.2% aged 80 years or older. A primary diagnosis of malignant disease was present in 5.7% of patients. With respect to medical interventions, 9.3% of participants were receiving home oxygen therapy or ventilatory support, 1.8% had peripheral or central venous catheters, and 13.0% had an indwelling urinary catheter or cystostomy. Overall, 13.2% of participants required at least one emergency home visit during the month preceding assessment. The most common primary diagnoses and the most prevalent coexisting chronic conditions are shown in Tables 2 and 3, respectively.

**TABLE 1:** Participants' characteristics and prevalence of emergency home visits (*N* = 506).

Characteristics	
Age in years, *n* (%)	
≤ 59	31 (6.1)
60–69	27 (5.3)
70–79	103 (20.4)
80–89	175 (34.6)
≥ 90	170 (33.6)
Sex, *N* (%)	
Male	206 (40.7)
Female	300 (59.3)
Malignancy as primary disease, *n* (%)	29 (5.7)
Home oxygen therapy or ventilatory support, *n* (%)	47 (9.3)
Peripheral or central venous catheters, *n* (%)	9 (1.8)
Indwelling urinary catheter or cystostomy, *n* (%)	66 (13.0)
Long‐term care need levels	
Lower (support need levels 1 and 2, or not certified^a^)	56 (11.1)
Middle (care need levels 1–3)	220 (43.5)
Higher (care need levels 4 and 5)	221 (43.7)
Missing	9 (1.8)
Visiting nurse use	366 (72.3)
Emergency home visit in the past month, *n* (%)	
Yes	67 (13.2)
No	420 (83.0)
Missing	19 (3.8)

^a^ “Not certified” indicates patients who had not applied for or had not been certified under the Japanese long‐term care insurance system.

**TABLE 2:** Ten most common primary diagnosis categories.

Primary diagnosis	*n* (%)
Dementia	93 (18.4)
Neurological diseases	70 (13.8)
Musculoskeletal conditions	69 (13.6)
Cerebrovascular disease	64 (12.6)
Cardiac diseases	45 (8.9)
Fracture‐related conditions	36 (7.1)
Malignancy	29 (5.7)
Chronic respiratory diseases	27 (5.3)
Arthritis or rheumatism	24 (4.7)
Digestive diseases	10 (2.0)

*Note:* Percentages are based on all included patients (*n* = 506) and do not sum to 100%. The 10 most common categories among the 18 predefined diagnosis categories are shown; 12 patients whose primary diagnosis did not fall within these categories are not listed.

**TABLE 3:** Ten most prevalent coexisting chronic conditions.

Coexisting chronic condition	*n* (%)
Hypertension	247 (48.8)
Dementia	202 (39.9)
Musculoskeletal conditions	189 (37.4)
Cardiac diseases	162 (32.0)
Urologic diseases	132 (26.1)
Cerebrovascular disease	121 (23.9)
Diabetes	117 (23.1)
Digestive diseases	103 (20.4)
Hyperlipidemia	103 (20.4)
Fracture‐related conditions	102 (20.2)

*Note:* Values are the number and percentage of patients with each coexisting chronic condition, based on all included patients (*n* = 506). Conditions are ordered by prevalence, and the 10 most prevalent are shown.

### 3.2 Prevalence of Chronic Conditions and Level of Multimorbidity

Descriptive statistics for the number of chronic conditions and the distribution of multimorbidity levels are presented in Table 4. Multimorbidity, defined as the presence of two or more chronic conditions, was observed in 479 of 506 participants (94.7%). Furthermore, 57.1% of patients had four or more chronic conditions. Consistent with classifications employed in prior studies, multimorbidity levels were categorized as low (2–3 chronic conditions) in 190 of 479 participants with multimorbidity (39.7%), medium (4–5 chronic conditions) in 42.4%, and high (≥ 6 chronic conditions) in 18.0%. In a sensitivity analysis excluding disuse syndrome, the prevalence of multimorbidity was similar (469/506, 92.7%; see Supporting Information for details).

**TABLE 4:** Prevalence of chronic conditions and levels of multimorbidity (*N* = 506).

Number of chronic conditions	*N*	%
Cumulative		
≥ 2 conditions	479	94.7
≥ 3 conditions	401	79.2
≥ 4 conditions	289	57.1
≥ 5 conditions	180	35.6
≥ 6 conditions	86	17.0
Exact number		
1 condition	27	5.3
2 conditions	78	15.4
3 conditions	112	22.1
4 conditions	109	21.5
5 conditions	94	18.6
≥ 6 conditions	86	17.0
Levels of multimorbidity (*N* = 479)		
Low (2–3 conditions)	190	39.7
Medium (4–5 conditions)	203	42.4
High (≥ 6 conditions)	86	18.0

*Note:* Cumulative values indicate the proportion of participants with at least the specified number of chronic conditions.

### 3.3 Associations Between Multimorbidity Level and Emergency Home Visits

Unadjusted and adjusted associations between multimorbidity level and emergency home visits are shown in Table 5. Using the low‐level multimorbidity group as the reference category, the adjusted odds ratio (aOR) for emergency home visits was 2.16 (95% CI, 1.09–4.31) in the medium‐level group and 2.72 (95% CI, 1.19–6.19) in the high‐level group. The ICC was 0.15 (range across imputed datasets, 0.09–0.17) in the unadjusted model and 0.16 (range, 0.10–0.18) in the adjusted model, indicating that approximately 15%–16% of the variability in emergency home visits was attributable to between‐facility differences. Model discrimination and other sensitivity analysis results are reported in the Supporting Information, including Figure S2 and Tables S2–S4.

**TABLE 5:** Associations between multimorbidity level and emergency home visits^a^ (*N* = 479).^b^

	Unadjusted	*p*	Adjusted^c^	*p*
	Crude OR (95% CI)		Adjusted OR (95% CI)	
Level of multimorbidity				
Low (2–3 conditions)	Reference		Reference	
Medium (4–5 conditions)	2.05 (1.05–3.98)	0.035	2.16 (1.09–4.31)	0.028
High (≥ 6 conditions)	2.57 (1.17–5.66)	0.017	2.72 (1.19–6.19)	0.017

Abbreviations: CI, confidence interval; OR, odds ratio.

^a^ Generalized linear mixed‐effects model with a logit link, including facility as a random intercept.

^b^ Patients with a single chronic condition (*n* = 27) were excluded from this analysis, consistent with prior literature (see Section 2).

^c^ Adjusted for age, sex, presence of a primary diagnosis of malignant disease, use of home oxygen therapy or ventilatory support, presence of peripheral or central venous catheters, and presence of an indwelling urinary catheter or cystostomy.

## 4 Discussion

In this multicenter study of patients receiving home care from primary care physicians in Japan, multimorbidity was highly prevalent, affecting 94.7% of participants. This prevalence exceeds that reported in prior Japanese studies while closely approximating estimates from international home care populations. The distribution of multimorbidity levels in the present study was likewise comparable to that observed in studies conducted in other countries. In addition, higher levels of multimorbidity were independently associated with an increased likelihood of emergency home visits.

### 4.1 Comparison With Prior Literature on Multimorbidity Prevalence in Home Care Settings

A marked discrepancy was observed between the prevalence of multimorbidity identified in this study and that reported in prior Japanese research. Kaneko et al. evaluated 250 Japanese home care patients and defined multimorbidity using chronic conditions categorized according to rubrics of the ICPC‐2 [15]. In that study, the reported prevalence of multimorbidity was 53.7%, substantially lower than the 94.7% observed here. The present study instead used a predefined list of 18 chronic condition categories informed by prior literature; a correspondence table mapping these categories onto ICPC‐2 rubrics (Table S1) showed close correspondence for most categories, although disuse syndrome, a component of the musculoskeletal conditions category and common among frail home care patients, had no identifiable ICPC‐2 counterpart. However, a sensitivity analysis excluding disuse syndrome yielded a similar prevalence of multimorbidity (92.7%), indicating that this measurement difference does not account for a substantial part of the discrepancy. A more likely explanation is how chronic conditions were identified. Kaneko et al. noted that conditions coded by physicians during home visits may have been underestimated, particularly in patients with communication difficulties such as dementia, and that coding per physician rather than per facility may have left some health problems unrecorded [15]. The present study, in contrast, identified conditions from a review of medical records, which is less likely to miss them.

Notably, the prevalence observed in this study closely aligned with international reports, including estimates of 92.1% in Canada [13] and 91.6% in Germany [14]. Furthermore, the distribution of chronic condition burden was comparable to Canadian data, in which 62.7% of patients had ≥ 4 chronic conditions compared with 57.1% in the present study [13].

### 4.2 Multimorbidity and Risk of Emergency Home Visits

The observed association between higher multimorbidity levels and increased likelihood of emergency home visits is consistent with extensive evidence linking multimorbidity to greater healthcare utilization [16]. Previous studies have demonstrated relationships between multimorbidity level and hospitalization risk as well as healthcare expenditures [17, 31]. Evidence from home care populations similarly indicates that higher levels of multimorbidity are associated with more frequent emergency department visits among patients with dementia [19]. Continuous, 24‐h access to home care providers has been emphasized by homebound patients and caregivers as a critical component of high‐quality home‐based primary care and is considered to reduce emergency department use and hospitalization [21, 32]. Beyond previously identified predictors of emergency home visits, such as malignancy and specific medical procedures [22, 23, 24], the present findings indicate that multimorbidity level is also associated with emergency home visit risk.

Count‐based multimorbidity level, as used in this study, is a simple but coarse measure of clinical burden. It does not capture the specific combinations of co‐occurring conditions studied in multimorbidity pattern research [27, 28], nor the broader dimensions of clinical complexity, such as functional impairment, psychosocial factors, and caregiving burden. Prior work using the Minnesota Complexity Assessment Method found that, among its domains, only the illness‐related domain was associated with unplanned home visits [33]. Multimorbidity level in the present study may partly reflect this illness‐related domain, although this could not be directly tested. Future studies using pattern‐ or complexity‐based approaches may build on the present findings to provide a fuller picture of clinical burden in this population.

### 4.3 Limitations

Several limitations merit consideration. First, the cross‐sectional design precludes inference of causal relationships and does not exclude reverse causation, although the acute conditions most commonly precipitating emergency home visits are not among the 18 predefined chronic condition categories, which likely limits this possibility. Second, the outcome was recorded as a dichotomous measure of any emergency home visit within the preceding month rather than as a count, precluding analysis of visit frequency. The one‐month window, chosen to limit the burden of medical record review for participating physicians, may not fully capture longer term healthcare utilization relative to longer periods such as 3 or 6 months, and this choice was not evaluated in a sensitivity analysis.

Third, the 18‐category list may not fully capture the morbidity profile. An international Delphi consensus study recommends including sensory impairment, anemia, and chronic pain [34], none of which were represented as discrete categories here. Other conditions common among homebound populations, such as skin conditions including pressure injury, were also not included as discrete categories. Future studies of multimorbidity in home care populations should consider incorporating these conditions. Fourth, disease severity and the broader dimensions of clinical complexity were not directly measured; the number of chronic conditions should therefore be interpreted as one dimension of multimorbidity rather than as a measure of overall clinical complexity. Fifth, reliance on physician‐reported chronic conditions and researcher classification of primary diagnoses introduces potential misclassification.

Sixth, patients excluded for unstable symptoms or a limited prognosis, together with those who died before the survey, are likely to have been at higher risk of emergency home visits. Their absence probably led to underestimation of the frequency of emergency home visits and may have biased the associations toward the null. Moreover, owing to feasibility constraints, the exact number of patients excluded for unstable symptoms or a limited prognosis could not be determined (Figure S1). Longitudinal studies that capture these patients are therefore needed. Seventh, the study was conducted within a limited geographic region, and most facilities were affiliated with a single university's training program—though one facility was an independent home‐care‐specialist clinic—which may limit generalizability.

In addition to these limitations, interpretation of the emergency home visit findings also warrants comparison with prior literature. As noted, fever and death certification are common indications for emergency home visits [15, 24]. In the present study, however, specific reasons for emergency visits—such as fever, dyspnea, falls, or end‐of‐life needs—were not collected, so the clinical circumstances underlying these encounters remain unclear; collecting this information should be a priority for future research in this population. Cohort studies incorporating death certification have reported an incidence of 1.61 emergency home visits per 10 person‐months [23], and that approximately 13.0% of patients experience emergency home visits at least monthly [22]. Although direct comparison is limited by methodological differences, the proportion of patients requiring emergency home visits in the present study (13.2%) was numerically similar.

### 4.4 Generalizability Across Home Care Delivery Systems

The findings should also be interpreted in the context of the Japanese home care system, in which physician‐led home visits constitute the principal model of medical care delivery. In contrast, several other health systems rely more on community‐based nursing services [35], while physician home visits are less common [36]. The observed association between multimorbidity level and emergency home visits may therefore partly reflect the physician‐led structure of Japanese home care, and further comparative studies across different systems are warranted to assess its applicability to nurse‐led models.

### 4.5 Strengths and Clinical Implications

Despite these limitations, several strengths should be emphasized. To current knowledge, this study is the first to demonstrate an association between multimorbidity level and emergency home visits among patients receiving physician‐led home care. Additionally, the observed prevalence of multimorbidity was consistent with international data, supporting the external relevance of the findings. These results may inform future efforts to design home care systems that better anticipate patients' care needs and risk of emergency home visits. Assessing multimorbidity level may, if confirmed in future studies, help identify individuals at elevated risk for emergency home visits and inform more proactive, needs‐based care planning within aging societies.

## 5 Conclusions

In this multicenter study of patients receiving physician‐led home care in Japan, multimorbidity was highly prevalent, affecting 94.7% of participants, a proportion comparable to estimates reported in international populations. Higher levels of multimorbidity were associated with an increased likelihood of emergency home visits. These findings suggest that greater multimorbidity may help identify patients at elevated risk of requiring emergency home services. Assessing multimorbidity level may, if confirmed in future studies, support more proactive, needs‐oriented home‐based care.

## Author Contributions

**Mitsuaki Ishii:** investigation, writing – review and editing. **Gen Nakayama:** conceptualization, investigation, methodology, formal analysis, writing – original draft, writing – review and editing. **Yu Sun:** investigation, writing – review and editing. **Noriyuki Kimura:** investigation, writing – review and editing. **Shoichi Masumoto:** funding acquisition, investigation, project administration, supervision, writing – review and editing. **Takashi Inaba:** writing – review and editing, investigation. **Sayaka Nin:** investigation, writing – review and editing. **Shuhei Hamada:** investigation, writing – review and editing. **Jun Hamano:** writing – review and editing. **Natsuki Kajikawa:** investigation, writing – review and editing.

## Funding

This work was supported by the Japan Society for the Promotion of Science, 21K15658.

## Ethics Statement

Ethics Committee of the Institute of Medicine, University of Tsukuba (approval no. 1951‐1).

## Consent

Informed consent, or consent via an opt‐out procedure, was obtained from participants as described in the Section 2 of the manuscript.

## Conflicts of Interest

The authors declare no conflicts of interest.

## Supporting information


**Figure S1:** Participant flow.

**Figure S2:** Dose–response relationship between chronic condition count and emergency home visits.
**Table S1:** Correspondence between the 18 chronic condition categories used in the present study and ICPC‐2 rubrics.
**Table S2:** Long‐term care need level by multimorbidity level (*N* = 479).
**Table S3:** Visiting nurse use by multimorbidity level (*N* = 479).
**Table S4:** Firth‐corrected odds ratios for emergency home visits by multimorbidity level.

## Data Availability

The data that support the findings of this study are available from the corresponding author upon reasonable request.
