# Supplementary material for: Multimorbidity Among Patients Receiving Home Care in Japan: Prevalence, Levels, and Association With Emergency Home Visits
Source: J Gen Fam Med. 2026 Aug 25;27(5):e70170. doi: 10.1002/jgf2.70170 (PMC13504458; doi:10.1002/jgf2.70170)
Supplement: Supplementary file 1 [file JGF2-27-e70170-s001.pdf]

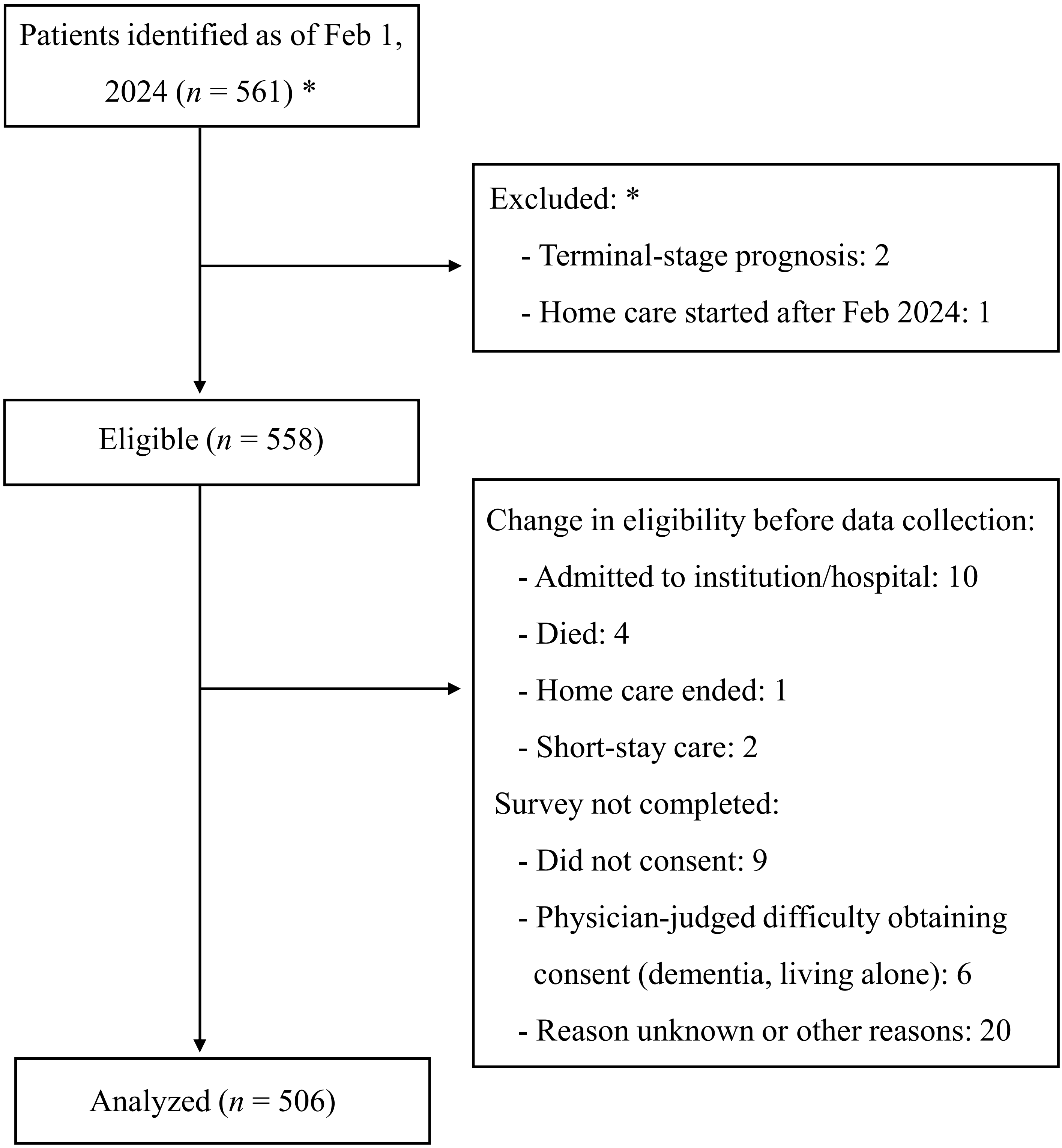

Supplementary Figure 1. Participant flow

* Each facility was originally asked to extract all patients receiving regular home visits as of February 1, 2024. Due to feasibility constraints, complete extraction may not have been achieved at every facility; as a result, more patients with terminal-stage prognosis are likely to have been excluded prior to compilation of these lists than the two identified here, although the exact number could not be determined. The count of 561 should therefore be regarded as a lower bound.
