# Supplementary material for: Multimorbidity Among Patients Receiving Home Care in Japan: Prevalence, Levels, and Association With Emergency Home Visits
Source: J Gen Fam Med. 2026 Aug 25;27(5):e70170. doi: 10.1002/jgf2.70170 (PMC13504458; doi:10.1002/jgf2.70170)
Supplement: Supplementary file 2 [file JGF2-27-e70170-s002.pdf]

Supplemental material

Supplementary Table S1. Correspondence between the 18 chronic condition categories used in the present study and ICPC-2 rubrics

Category in present study (18-category list, full example set)	ICPC-2 rubric code(s)	ICPC-2 rubric label (WONCA)	Note
1. Hypertension	K86<br>K87	Hypertension uncomplicated<br>Hypertension complicated	
2. Diabetes	T89<br>T90	Diabetes insulin dependent<br>Diabetes non-insulin dependent	
3. Hyperlipidemia	T93	Lipid disorder	
4. Cerebrovascular disease	K91	Cerebrovascular disease	
5. Cardiac disease (e.g., ischemic heart disease, heart failure, atrial fibrillation, PAD)	K74<br>K76<br>K77<br>K78<br>K92	Ischaemic heart disease w. angina<br>Ischaemic heart disease w/o angina<br>Heart failure<br>Atrial fibrillation/flutter<br>Atherosclerosis/PVD	
6. Chronic respiratory diseases (e.g., asthma, COPD, chronic bronchitis)	R96<br>R95<br>R79	Asthma<br>Chronic obstructive pulmonary disease<br>Chronic bronchitis	
7. Digestive diseases (e.g., GERD, gastric ulcer, hepatitis, liver cirrhosis)	D84<br>D86<br>D72<br>D97	Oesophagus disease<br>Peptic ulcer other<br>Viral hepatitis<br>Liver disease NOS	
8. Kidney disease (e.g., chronic kidney disease)	U88 or U99	Glomerulonephritis/nephrosis<br>Urinary disease, other	

**Category in present study (18-category list, full example set)**	**ICPC-2 rubric code(s)**	**ICPC-2 rubric label (WONCA)**	**Note**
9. Urologic diseases (e.g., prostatic hypertrophy, overactive bladder, neurogenic bladder)	Y85<br>U02	Benign prostatic hypertrophy<br>Urinary frequency/urgency	Overactive bladder and neurogenic bladder have no dedicated diagnosis rubric.
10. Arthritis or rheumatism (e.g., osteoarthritis, rheumatoid arthritis)	L88<br>L89<br>L90<br>L91	Rheumatoid/seropositive arthritis<br>Osteoarthrosis of hip<br>Osteoarthrosis of knee<br>Osteoarthrosis other	
11. Musculoskeletal conditions (e.g., disuse syndrome, lumbar spinal canal stenosis)	L86	Back syndrome with radiating pain	L86 is the closest available rubric for lumbar spinal canal stenosis, based on the defining symptom of radiating pain, rather than a rubric for the structural diagnosis itself. Disuse syndrome has no identifiable corresponding rubric
12. Fracture-related conditions (e.g., femoral neck fracture, spinal cord injury)	L75<br>N81	Fracture: femur<br>Injury nervous system other	
13. Osteoporosis	L95	Osteoporosis	
14. Dementia	P70	Dementia	
15. Neurological diseases (e.g., Parkinson disease, epilepsy)	N87<br>N88	Parkinsonism<br>Epilepsy	
16. Mental disorders (e.g., depression, anxiety)	P74<br>P76	Anxiety disorder/anxiety state<br>Depressive disorder	
17. Endocrine disorders (e.g., thyroid disease)	T85<br>T86	Hyperthyroidism/thyrotoxicosis<br>Hypothyroidism/myxoedema	

Category in present study (18-category list, full example set)	ICPC-2 rubric code(s)	ICPC-2 rubric label (WONCA)	Note
18. Malignancy	A79, B72–B74, D74–D77, F74, H75, K72, L71, N74, R84–R85, S77, T71, U75–U77, W72, X75–X77, Y77–Y78	Malignancy NOS / site-specific malignant neoplasm rubrics	No single corresponding rubric; ICPC-2 classifies malignancy by primary anatomical site

Note: ICPC-2, International Classification of Primary Care, second edition (WONCA International Classification Committee). Correspondence was based on the full ICPC-2 rubric list (686 rubrics). Where the category description included illustrative examples (“e.g.”), only those named conditions were mapped.

Handling of missing outcome data in multiple imputation

Missing values in the outcome variable, as well as in covariates, were imputed using the same fully conditional specification model. The outcome was included as a predictor in the imputation model for other variables, consistent with recommendations that the outcome be included in the imputation model to preserve its association with other variables. We acknowledge that imputing missing values

in the outcome itself is a debated practice; in our case, missingness in the outcome was limited (19 of 506 patients, 3.8%), and we considered the potential bias from excluding these patients (through complete-case analysis) to outweigh the concerns associated with outcome imputation.

Model discrimination

Model discrimination was assessed using the C-statistic (area under the receiver operating characteristic curve). The C-statistic was calculated separately for each of the 20 imputed datasets and averaged.

The adjusted model showed a mean C-statistic of 0.76 (range across imputed datasets, 0.74–0.78). This indicates acceptable discrimination.

Sensitivity analyses

1. Sensitivity analysis additionally adjusted for long-term care need level and use of home-visit nursing services

Long-term care need level and visiting nurse use were not included in the primary model, for the reasons described in the Methods (long-term care need level may represent a consequence of

multimorbidity, and visiting nurse use is largely determined by long-term care need level). We therefore evaluated them separately, in a sensitivity analysis in which the primary adjusted model was repeated with these two variables added as additional covariates (*N* = 479). The estimates were similar to those of the primary analysis, with aOR of 2.00 (95% CI, 1.00–3.99) for the medium-level group and 2.69 (95% CI, 1.19–6.08) for the high-level group.

To help readers assess the extent to which long-term care need level and visiting nurse use may reflect the consequences of multimorbidity, we additionally show their distributions across multimorbidity levels (Supplementary Tables S2 and S3).

Supplementary Table S2. Long-term care need level by multimorbidity level (*N* = 479)

Long-term care need levels	2–3 conditions	4–5 conditions	≥6 conditions
Lower (support need levels 1 and 2, or not certified)	29 (15.6%)	16 (8.0%)	6 (7.1%)
Middle (care need levels 1–3)	73 (39.2%)	96 (47.8%)	39 (45.9%)
Higher (care need levels 4 and 5)	84 (45.2%)	89 (44.3%)	40 (47.1%)
Missing (n = 7)			

Supplementary Table S3. Visiting nurse use by multimorbidity level (*N* = 479)

Visiting nurse use	2–3 conditions	4–5 conditions	≥6 conditions
No	38 (20.0%)	73 (36.0%)	23 (26.7%)
Yes	152 (80.0%)	130 (64.0%)	63 (73.3%)

2. Sensitivity analysis excluding disuse syndrome

To exclude disuse syndrome as far as the data structure allowed, we applied a three-step procedure. First, for participants whose primary diagnosis included the term “disuse syndrome” and who had not been checked for comorbidity category 11 (musculoskeletal conditions), category 11 was recoded as 0. Second, when the primary diagnosis clearly indicated a different musculoskeletal condition, such as lumbar spinal canal stenosis, category 11 was recoded as 1. Third, when the primary diagnosis was unrelated to musculoskeletal conditions (e.g., Alzheimer's disease) but category 11 had been checked, category 11 was conservatively treated as absent, because it could not be determined whether the checked comorbidity represented disuse syndrome or another musculoskeletal condition.

Under this approach, multimorbidity was observed in 469 of 506 participants (92.7%). Among participants with multimorbidity, levels were categorized as low (2–3 chronic conditions) in 215 (45.8%), medium (4–5 chronic conditions) in 193 (41.2%), and high (≥6 chronic conditions) in 61 (13.0%).

3. **Sensitivity analysis using restricted cubic splines**

As suggested by the reviewer, we examined the relationship between chronic condition count and emergency home visits without using fixed cutoffs. We re-fit the adjusted model with chronic condition count included as a smooth curve (restricted cubic spline, 3 degrees of freedom, using the *splines* package in R) instead of a category. Multiple imputation and model fitting were performed in the same way as in the primary analysis.

For each value of chronic condition count (2 to 11), we calculated the predicted odds ratio compared with patients having 2 conditions, while keeping the other characteristics the same. The predicted odds ratio increased as the number of chronic conditions increased, reaching its highest point at 6 conditions (OR 3.19, 95% CI 1.23–8.29). The 95% confidence interval did not cross 1 for patients with 5 or 6 conditions, but did cross 1 for other values. For patients with 8 or more conditions, the estimates should be interpreted with caution because very few patients had these high counts (8 or fewer patients per value).

Overall, the pattern of increasing predicted odds ratios with increasing chronic condition count was broadly consistent with the primary analysis, although statistical significance was reached only for 5 and 6 conditions, likely reflecting the limited number of patients at each individual value. While this analysis does not provide conclusive statistical confirmation at every point, the overall pattern does not contradict the primary categorical findings.

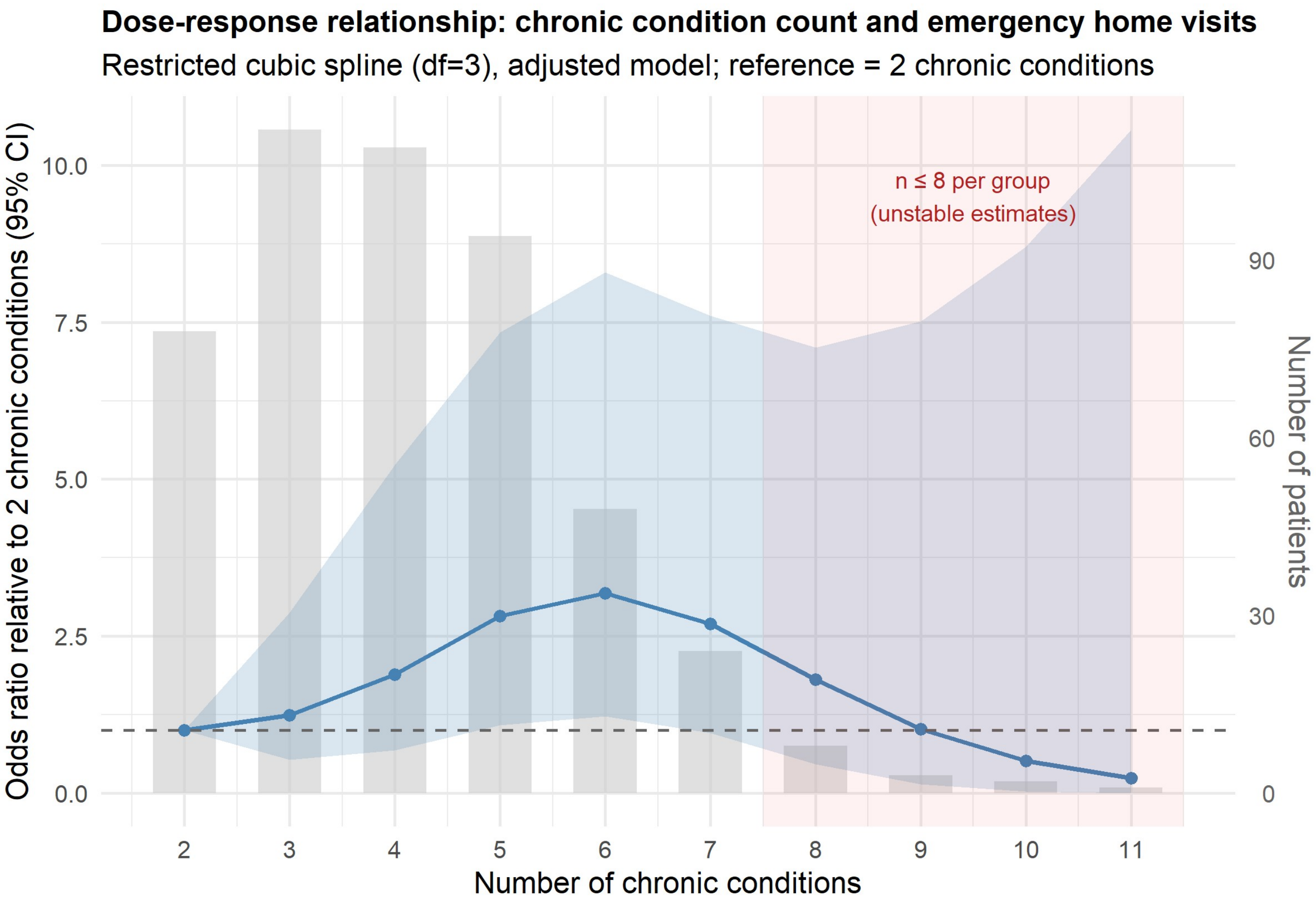

Supplementary Figure 2. Dose-response relationship between chronic condition count and emergency home visits.

4. Sensitivity analysis using Firth's penalized likelihood method

As suggested by the reviewer, we performed a sensitivity analysis using Firth's penalized likelihood method to assess the robustness of the primary findings. This method reduces small-sample bias in logistic regression. Firth's method was implemented using the *logistf* package in R. Standard implementations of Firth's method, including *logistf*, support fixed-effects models only. They cannot include random effects. For this reason, facility could not be included as a random intercept in this sensitivity analysis. This analysis therefore does not adjust for clustering of patients within facilities. This is a limitation of the sensitivity analysis. The same 20 multiply imputed datasets used in the primary analysis were used here. Firth's penalized logistic regression was fitted separately to each of the 20 datasets, using the same exposure and covariates as the primary model.

The *mice::pool()* function does not support *logistf* model objects. Estimates from the 20 datasets were therefore pooled manually using Rubin's rules. Odds ratios and 95% confidence intervals were calculated from the pooled log-odds estimates and their combined variance.

Results of the Firth-corrected sensitivity analysis were similar to those of the primary analysis. This supports the robustness of the main findings (Supplementary Table S4).

Supplementary Table S4. Firth-corrected odds ratios for emergency home visits by multimorbidity level

	Unadjusted<br>Crude OR (95% CI)	*P* value	Adjusted<br>Adjusted OR (95% CI)	*P* value
**Level of multimorbidity**				
Low (2–3 conditions)	Reference		Reference	
Medium (4–5 conditions)	2.18 (1.10–4.32)	0.028	2.26 (1.13–4.51)	0.023
High (≥6 conditions)	2.89 (1.30–6.40)	0.012	2.96 (1.31–6.68)	0.012
